# Supplementary material for: Hypoxia favors tumor growth in colorectal cancer in an integrin αDβ1/hemoglobin δ-dependent manner
Source: Life Sci Alliance. 2024 Dec 3;8(2):e202402925. doi: 10.26508/lsa.202402925 (PMC11629678; doi:10.26508/lsa.202402925)
Supplement: Supplementary file 1 [file LSA-2024-02925_SdataFS1.pdf]

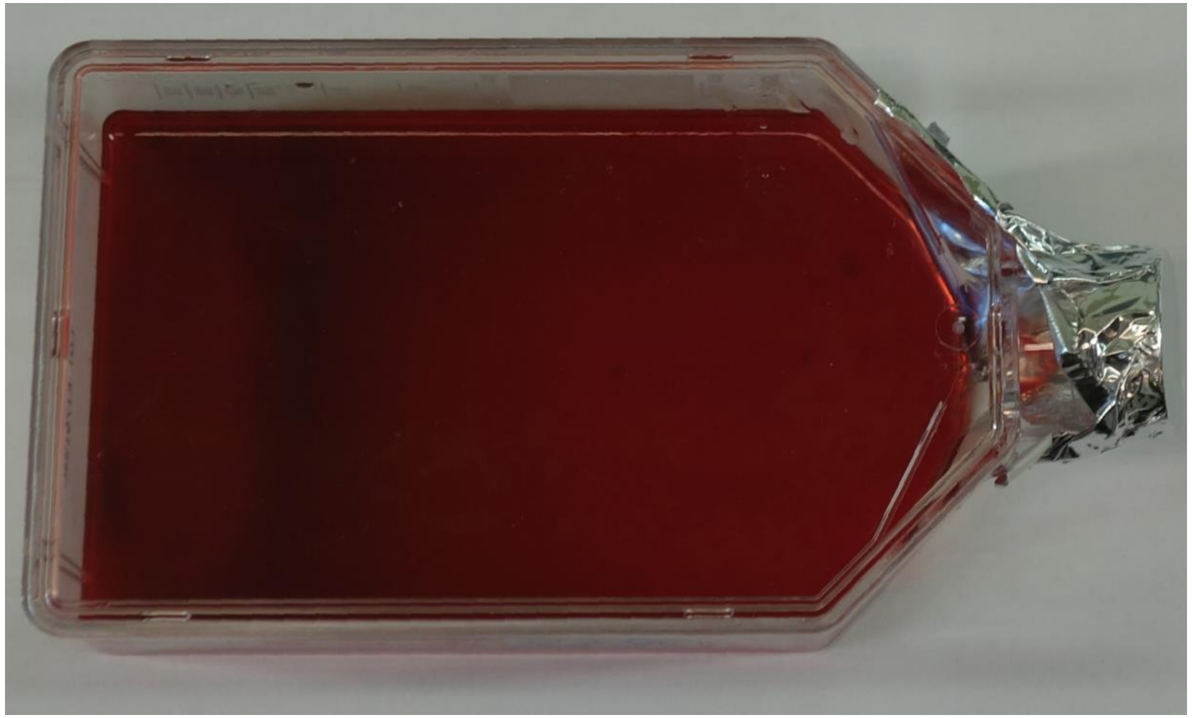

Source Data Suppl. Fig.S1. Formation of an oxygen gradient in whole blood-tumor cell co-culture. Colo205 cells were cultured in 10% whole blood/RPMI overnight with the bottle cap film-sealed (right) in hypoxia chamber. Purple-colored red blood cells form in oxygen-depleted environment (left).
