# Supplementary material for: Hypoxia favors tumor growth in colorectal cancer in an integrin αDβ1/hemoglobin δ-dependent manner
Source: Life Sci Alliance. 2024 Dec 3;8(2):e202402925. doi: 10.26508/lsa.202402925 (PMC11629678; doi:10.26508/lsa.202402925)
Supplement: Supplementary file 2 [file LSA-2024-02925_SdataF1.1.pdf]

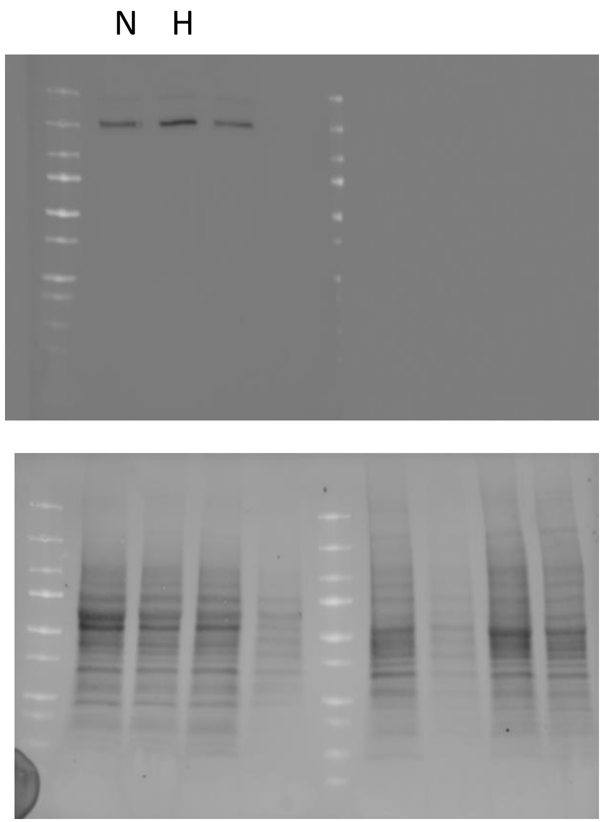

Protein staining

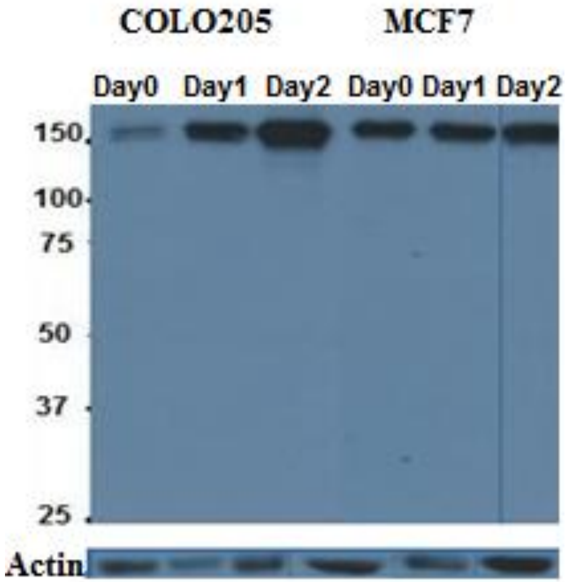

Source data for Fig.1C. Examples of αD immunoblots and protein staining of normoxic (N) and hypoxic (H) COLO205 cells.
