# Supplementary material for: Hypoxia favors tumor growth in colorectal cancer in an integrin αDβ1/hemoglobin δ-dependent manner
Source: Life Sci Alliance. 2024 Dec 3;8(2):e202402925. doi: 10.26508/lsa.202402925 (PMC11629678; doi:10.26508/lsa.202402925)
Supplement: Supplementary file 6 [file LSA-2024-02925_SdataF3.2.pdf]

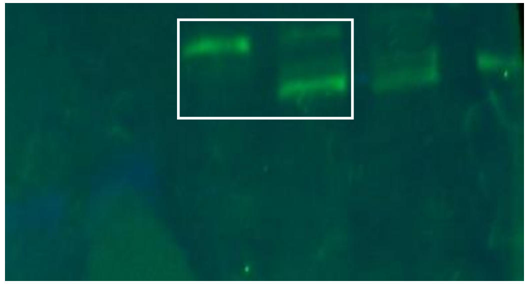

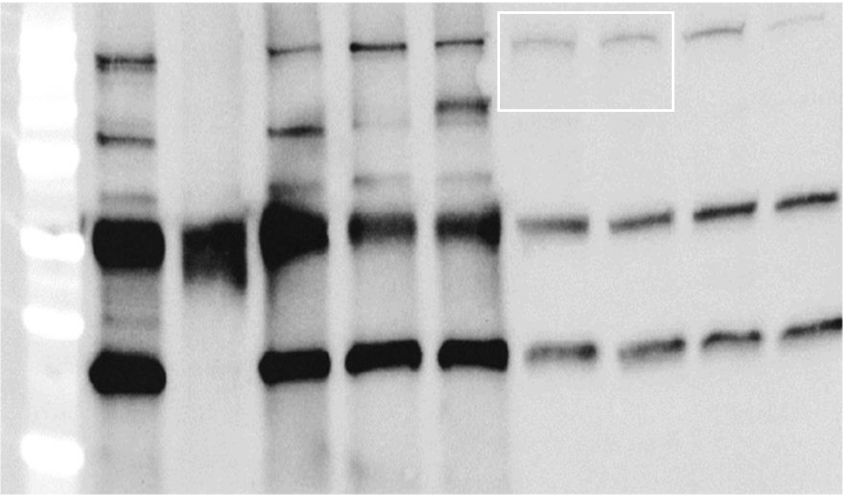

Source data for Fig.3A. COLO205 and U937 cells were sequentially blotted with β1, β2 and αD integrin IgG. Immunoprecipitation (IP) was with αD integrin IgG or control IgG.
