# Supplementary material for: Hypoxia favors tumor growth in colorectal cancer in an integrin αDβ1/hemoglobin δ-dependent manner
Source: Life Sci Alliance. 2024 Dec 3;8(2):e202402925. doi: 10.26508/lsa.202402925 (PMC11629678; doi:10.26508/lsa.202402925)
Supplement: Supplementary file 7 [file LSA-2024-02925_SdataF3.3.pdf]

Colo IgG Colo IP.β1 LS174T IP. β1 Colo lysate

1 2 3 4 5 6 7 8

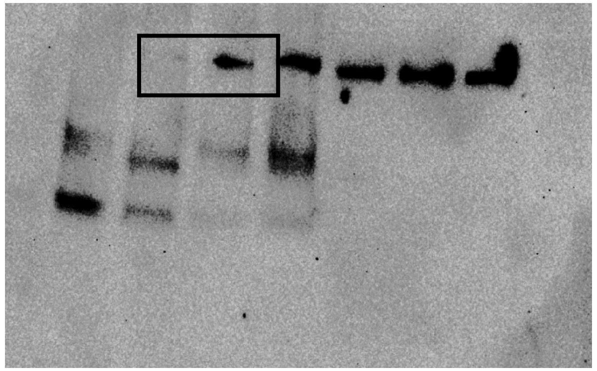

WB: αD (ref. Figure 3b)

Colo IgG Colo IP.β1 LS174T IP. β1 Colo lysate

1 2 3 4 5 6 7 8

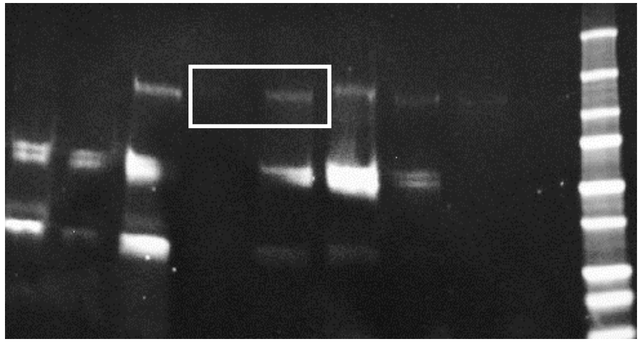

WB: β1 of above gel

Source data for Fig.3B. COLO205 and LS174T cells were immunoprecipitated (IP) with β1 integrin or control IgG and blotted for αD (above) or β1 integrin (below).
